# Intellectual Disability and Blended Phenotypes: Insights from a Centre in North India

**DOI:** 10.1155/2024/6009569

**Published:** 2024-08-22

**Authors:** Inusha Panigrahi, Sudha Rao, Shalu Verma Kumar, Divya Kumari, Parminder Kaur

**Affiliations:** ^1^ Department of Pediatrics APC PGIMER, Chandigarh, India; ^2^ Dhitiomics Technologies Private Ltd., Bangalore, India

## Abstract

Intellectual disability (ID) is seen in around 2.5% of global population and can vary from mild to severe and profound ID. There can be multiple affected family members if it is inherited, though many autosomal dominant ID cases would be due to de novo mutations are very less likely to recur in families. A confirmatory diagnosis is facilitated by genetic testing like chromosomal microarray and next generation sequencing. We describe here our cohort of 15 patients: children and adolescents with ID diagnosed by using sequencing technologies and parental segregation studies. Most of the variants identified were de novo variants and consistent with sporadic occurrence, and blended phenotypes were identified. Appropriate genetic counseling was performed and options for prenatal diagnosis were discussed. Thus, advanced sequencing technologies enable identification of likely causative de novo variants associated with intellectual disability and dysmorphism.

## 1. Introduction

Intellectual disability is a clinically and genetically heterogenous group with multiple tests being used to reach a definitive diagnosis in complicated cases [1–3]. The presentations include developmental delay, autism, seizures, behavioral problems, and magnetic resonance imaging (MRI) abnormalities in certain cases. With availability of high-throughput technologies, more cases are getting molecular confirmation and better prognostication. The traditional Sanger sequencing using fluorescently labelled terminating nucleotides followed by gel electrophoresis has been the gold standard for sequencing. The major advantage of new “second generation sequencing” (SGS) technology is considerably high throughput and hence low cost per base sequenced. In the SGS technology developed by Illumina, adapter-ligated DNA sequences are amplified directly in the flow cell subsequently utilised for sequencing. In SOLiD (sequencing with oligonucleotide ligation and detection) technologies, adapter-ligated fragments are hybridized to beads on glass slide, coated with an oligonucleotide complementary to one of the adapters and used for amplification in a water-in-oil emulsion polymerase chain reaction (PCR). Thus, next generation sequencing (NGS) is being increasingly used for confirmation of diagnosis in phenotypes with genetic heterogeneity. We report some Indian patients with intellectual disability identified on NGS based testing.

## 2. Methods

This is an analysis of the patients seen in the specialty clinic of tertiary care centre. The main objective was to find variants especially de novo single nucleotide variants (SNVs) related to ID and phenotypic variability in Asian Indians. The clinical evaluation was done as per standard protocols including X-rays of the dorso-lumbar spine and wrist for identifying vertebral abnormalities, dysostosis, and advanced or delayed bone age. Ultrasonography of abdomen was done to identify any renal anomalies, and echocardiography was done to find out cardiac anomalies. In unexplained ID, basic metabolic work up like plasma ammonia, serum lactate and creatine kinase levels were performed. The inclusion criteria included (1) presence of developmental delay, microcephaly, and/or behavioural abnormalities; (2) negative results on karyotyping and multiplex ligation probe amplification (MLPA) and/or chromosomal microarray testing not showing copy number variants; (3) referred to clinic for dysmorphism or other anomalies; and (4) MRI not suggestive of structural anomalies or neurometabolic disorder.

NGS for targeted genes causing intellectual disability or autism was done as per standard protocols using variant filtering, prioritization, etc. Variant classification was done as per American College of Medical Genetics and Genomics (ACMG) guidelines [4]. Pretest and posttest genetic counseling was offered in the families. Ethical clearance was obtained from the Institute ethics committee. Written informed consent was taken from the parents for genetic testing, and for photographs from all the participants, and Indian Council of Medical Research (ICMR) ethical guidelines were followed.

## 3. Results

**Figure 1:**
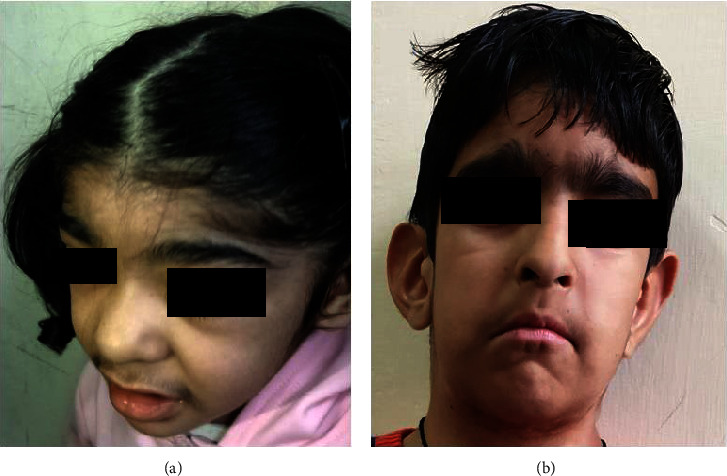
Facial features showing mild flat facies and prominent cheeks and open mouth in child with PURA syndrome (a and b).

This study encompasses fifteen patients with ID, in a majority of whom heterozygous variants were identified using targeted NGS. MRI brain was done in Case 2 (Figure 1) at 1 year age and showed nonspecific altered signal intensities and a small arachnoid cyst. Figure 1 depicts facial profile of patients with Coffin Siris syndrome–patients 2 and 13 in Table 1. Table 1 lists the clinical details and variants found in these patients.  Figure 1S shows Sanger depicting variant in Case 2. Four of these cases are described in detail below:

1-year-old female child presented with developmental delay and seizures. Examination revealed plagiocephaly, squint, prominent cheeks, poorly formed nasal columella, inverted V-shaped upper lip, open mouth (Figure 1), and hypotonia. She also had stranger anxiety. The creatine phosphokinase level was 348.5 units (normal level < 228 units), and MRI brain was normal. The thyroid stimulating hormone (TSH) level was 2.58 *μ*IU/ml (normal: 0.7–5.97 units). NGS was done to cover genes causing hypotonia also including congenital myopathy genes. It revealed an exon1 variant in the *PURA* gene. The heterozygous nonsense variant identified was *PURA*: c.791G > A: p. (Trp264^∗^). In addition, there were compound heterozygous missense variants in the *HERC2* gene: p.Asn370Ser and p.Val2532Leu in exon 10 and exon 47 of the gene.

**Figure 2:**
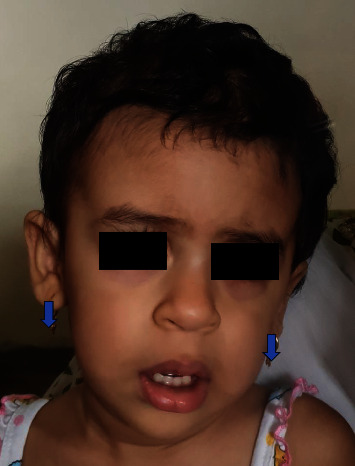
The facial dysmorphism including coarse facies and hirsutism in child with Coffin Siris syndrome with pathogenic *ARID1B* variant.

A two-year-old girl was brought to the outpatient clinic with global developmental delay and hyperactivity. There was no positive family history and no consanguinity. Examination revealed short stature, triangular facies, microcephaly, coarse facies, bushy eyebrows, anteverted nares, clinodactyly, and generalised hirsutism. Some of the features like microcephaly and short stature were consistent with Rubinstein-Taybi syndrome (RSTS) whereas coarse facies, thick eyebrows, and hirsutism were suggestive of possible chromatinopathy like KBG syndrome. The targeted NGS testing revealed two pathogenic variants associated with ID in *ANKRD11* and *EP300* genes (Table 1), confirming a blended phenotype.  Figure 2 depicts facial profile of patient with Coffin Siris syndrome—patient 2 in Table 1.

**Figure 3:**
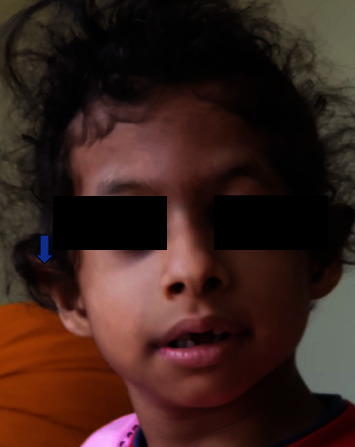
Facial profile of Case 15 with possible blended phenotype.

A three-year-old female child was brought with features of seizures, growth deficiency, cardiac defect, and autism. Examination revealed microcephaly and dysmorphism (Figure 3). There were no renal anomalies on ultrasonography. Testing for chromosomal deletions and duplications was negative. Targeted next generation sequencing revealed *GABRA2* and *FLNA* variants. The facies was consistent with that associated with *FLNA* gene variant, but further characterisation needed.

A 3-year-old male child (case 9) presented with low birth weight (2 kg), developmental delay, microcephaly (−3.4Z), right microcornea, long eyelashes, squint, retrognathia, low-set ears hearing impairment, and a happy demeanor (Figure 2S). Echocardiography revealed 4 mm patent ductus arteriosus (PDA), with left to right shunt. MLPA testing for chromosomal deletions and duplications was negative. NGS testing revealed a likely pathogenic heterozygous variant in *PACS1*: c.607C > T(p.Arg203Trp).

## 4. Discussion

Children with ID can have variable presentations, and some have additional phenotypic abnormalities which aid in the diagnosis. Having a child with severe ID in family can create management issues in the family and can be a burden on the public health system especially if additional systemic abnormalities are also present like cardiac or renal anomalies. The usual first line test in unexplained developmental delay or ID with or without dysmorphism is chromosomal microarray (CMA) for deletions and duplications [5]. However, those negative on CMA or having characteristic features of a particular syndrome like Coffin Siris syndrome can be further tested by NGS. MLPA for common deletions or duplications is another cheaper alternative test used for known chromosomal microdeletions or duplications. Nowadays, targeted NGS is being increasingly used for diagnosis of genetic disease, unexplained phenotypes, and for gene expression profiling and genome research.

Intellectual disability can be very heterogenous with overlapping phenotypes. “Overlapping phenotypes” refer to presentation with developmental delay, behavioural abnormalities, with or without seizures or stereotypies in several neurodevelopmental disorders. Few have additional malformations, and MRI brain can show nonspecific abnormalities. In selected cases, a facial gestalt can help in making a clinical diagnosis. Sometimes additional features can help in identifying a particular syndrome as in case of patient 4 and patient 10 in present report. We identified some novel variants in known genes causing ID in the Asian Indians. A variant of unknown significance as per ACMG guidelines can be intriguing and further evaluation becomes important. A parental segregation analysis and use of bioinformatic tools for predicting effect of the variants are being increasingly used for finding out the relevance of the variants identified.

In present analysis, most variants were heterozygous mutations mostly de novo; and 9 variants were pathogenic or likely pathogenic variants. Case 1 showed a heterozygous variant *MECP2* (NM_001110792.2): c.352C > T (p.Arg118Trp), rs28934907, located at ChrX:153297719 (GRCh37); pathogenic as per ACMG guidelines, causes Rett Syndrome and is extensively published [6]. The syndrome has X-linked dominant (XLD) inheritance including atypical and preserved speech variants [OMIM #312750]. Another girl in present series with ID and stereotypic hand movements also showed *MECP2* gene variant, which has also earlier been reported with patients diagnosed with Rett syndrome.

Case 2 showed a heterozygous null variant (frame-shift) NM_001374820: c.5467delG, in gene *ARID1B*. It is autosomal dominant disorder and associated with Coffin-Siris syndrome type1 [OMIM #135900] [7]. This variant is not found in gnomAD exomes or gnomAD genomes but has been reported at ClinVar and classified as “Pathogenic” in association with Coffin-Siris syndrome [Accession: SCV001736716.1].

Case 3 showed a heterozygous variant NM_001104631.2: c.701G > A, rs766490621, in *PDE4D* gene. This VUS is located at Chr5:58481072 (GRCh37). It is reported across population databases with the following frequencies: *T* = 0.000016 (4/248010, GnomAD_exome); *T* = 0.000033 (4/119640, ExAC); and *T* = 0.00003 (1/32060, ALFA).

The *MYT1L* gene variant in Case 4 is a heterozygous SNV: NM_015025.4: c.1700G > A, rs878853045. It is located at Chr2:1915795 (GRCh37). It is reported at ClinVar [Accession: VCV000235469.8] as “likely pathogenic” in association with autosomal dominant ID. It has previously been reported as a de novo variant in a patient with intellectual disability, plagiocephaly, 5th finger clinodactyly, an ataxic gait, hyperphagia, and cerebral atrophy [reported as p.Arg569Gln] [8].

A male child with ID (Case 5) showed a heterozygous variant NM_001256182.2: c.7814T > G, rs1131691512, in *ANKRD11* gene. It is located at Chr16: 89335064 (GRCh37). It is reported at ClinVar [Accession: VCV000429654.4]. This variant is not found in gnomAD exomes or gnomAD genomes. Macrodontia involving upper central incisors is a diagnostic hallmark of KBG syndrome, which is named using initials of first described patients with the syndrome. This condition can also result from microdeletion of chromosome 16q24 in some patients, a copy number variant (CNV), which can be identified on chromosomal microarray [9].

Interestingly, in Case 6 with coarse facies, a heterozygous null variant (frame-shift) was identified NM_015570.4: c.416_419delAGAG, in gene *AUTS2*. This likely pathogenic variant is associated with autism spectrum disorder due to AUTS2 deficiency. Case 7 showed a hemizygous deletion chrX-67273549-GGGAGTAGCT (10 bp) (*OPHN1*: p.A752Kfs^∗^82). This variant NM_002547.3: c.2253_2262delAGCTACTCCC was not found in gnomAD exomes or gnomAD genomes and not reported at ClinVar; thus the variant was labelled as “likely pathogenic.” *OPHN1*-related ID is a X-linked recessive disorder affecting males [10].

**Figure 4:**
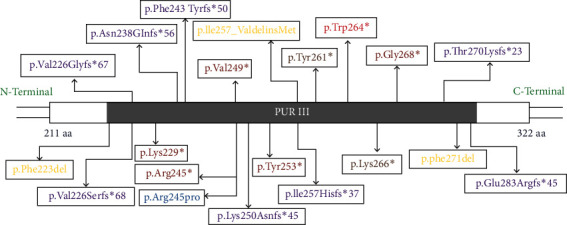
A diagrammatic representation of the variants identified in the PURA gene causing PURA syndrome.

Case 8 showed a heterozygous missense variant, a novel likely pathogenic variant_ NM_005859.5: c.791G > T (p.Trp264^∗^) in the *PURA* gene. *PURA* gene codes for PUR-alpha, which is a purine-rich protein and essential for normal brain development including oligodendrocytes and astrocytes. It is in exon 3 of transcript ENSG00000185129.5 for gene *PURA* [GTExVersion: v8] in the hotspot region of the gene. In a large cohort study by Reijnders et al., four variants in *PURA* gene were identified as recurrent variants which include p.Lys97Glu, p. Phe233del, p.Arg245Pro, and p.Phe271del. This disorder has some similarities to cutis laxa and myopathic facies, gait abnormalities and increased creatine kinase suggest muscle involvement in PURA syndrome [11, 12]. The pathogenic and likely pathogenic variants in the *PURA* gene product, in domain III have been illustrated in Figure 4. A recent article has identified high frequency of de novo mutations or variants (DNMs) in few genes which include *PURA, SATB2, SCN1A*, and *TUBA1A* and these are mostly found in cases of neurodevelopmental disorders (NDDs) [13].

Case 9 showed a heterozygous variant, NM_018026.4(*PACS1*): c.607C > T, rs398123009, is located at 11: 65978677 (GRCh37). It is reported at ClinVar [Accession: VCV000039581.39] where it is classified as “pathogenic:” in association with Schuurs–Hoeijmakers syndrome [14]. The nucleotide position is conserved across 35 mammalian species (GERP RS: 4.79). Blended phenotype was observed in Case 10, a 2-year-old girl with ID. She showed a heterozygous frameshift variant on NGS: NM_001256183.2 (*ANKRD11*): c.1388_1389del (p.Lys463Argfs^∗^29). A “Likely pathogenic” variant causing KBG syndrome. This patient also showed a heterozygous null variant, *EP300* (NM_001429.4): c.4261dup (p.Tyr1421Leufs^∗^22); associated with Rubinstein-Taybi syndrome (RSTS), a novel pathogenic variant as per ACMG guidelines. Variants in *EP300* have also been reported in association with colorectal cancer, and Menke–Hennekam syndrome 2. It is not found in gnomAD exomes or gnomAD genomes. Thus, the NGS testing enabled identification of a “blended” phenotype and hence enabled appropriate genetic counseling. When different variants are causing similar phenotypes, sometimes it may be difficult to differentiate which clinical feature is due to which variant, thus the phenotype is mixed or blended.

Similarly, in case 15, a single variant could not explain the full phenotype. Some features were explained by the FLNA gene reverse phenotyping; but autism and seizures could be explained by the *GABRA2* variant; thereby suggesting possibility of a blended phenotype. The FLNA variant c.6737C > T (p.Pro2246Leu), was not found in ExAC and 1000 genomes database, and classified as likely pathogenic using in-silico tools and ACMG guidelines, predicted deleterious on PROVEAN, LRT, and REVEL databases. Franklin automated classification classified it as VUS, leaning towards pathogenic. The *GABRA2* variant in this child has earlier been reported as VUS on ClinVar.

The heterozygous variant in Case 11 NM_001204398.1 (*PITX2*): c.185–1477C > G, rs1578453824, located at Chr4:111544002 (GRCh37). It is neither reported at ClinVar nor in gnomAD exomes or gnomAD genomes. This gene has been earlier associated with ring dermoid which was seen in the child [15]. Thus, the variant was a variant of unknown significance (VUS). Confirmation of SNVs and parental segregation is done by Sanger sequencing. Nowadays, copy number variants (CNVs) can also be identified on NGS analysis by using specialised tools or pipelines due to availability of better technologies or tools using artificial intelligence. Severe intellectual disability with microcephaly and multiple malformations is more likely due to chromosomal cause and hence CMA is first line genetic testing [5].

Thus, we found some known and novel variants in children with ID. Hence, to conclude, NGS enables finding mutations in known genes with relative ease, thus facilitating appropriate genetic counseling in the affected families. It is a useful adjunct to MLPA and CMA to make definitive clinical diagnosis, in patients with mild to moderate intellectual disability.

## Figures and Tables

**Table 1:** Clinical and variant description in the ID patients in present report.

Sl. no.	Age/Sex	Clinical features	Diagnosis	Gene	Zygosity	HGVSc	HGVSp	Variant classification
1	4 year/F	Developmental delay, seizures, microcephaly, hypotonia, epicanthic folds, brachydactyly	Rett syndrome	*MECP2*	Heterozygous	NM_001110792.2: c.352C > T	NP_001104262.1: p.Arg118Trp	Pathogenic
2	5 year/F	Microcephaly, developmental/speech delay, hypotonia, hirsutism, preauricular sinuses, short little fingers, pubic hairs	Coffin Siris syndrome	*ARID1B*	Heterozygous	NM_001374820: c.5467delG	NP_065783.3: p.Gly1824ValfsTer27	Likely pathogenic
3	4 year/F	ID, obesity, abnormal behaviour	Dominant ID	*PDE4D*	Heterozygous	NM_001104631.2: c.701G > A	NP_001098101.1: p.Arg234Gln	VUS
4	6 year/M	Developmental delay, microcephaly, behavioural problems, bilateral peritrigonal hyperintensities	Dominant ID	*MYT1L*	Heterozygous	NM_015025.4: c.1700G > A	NP_055840.2: p.Arg567Gln	Likely pathogenic
5	13 year/M	ID, hyperactivity, large ears, bulbous nose, broad eyebrows, hemivertebrae (L4-L5)	KBG syndrome	*ANKRD11*	Heterozygous	NM_001256182.2: c.7814T > G	NP_001243111.1: p.Leu2605Arg	Likely pathogenic
6	5 year/F	Coarse facies, autism, intellectual disability, no malformations	Sporadic ID	*AUTS2*	Heterozygous	NM_015570.4: c.416_419delAGAG	NP_056385.1: p.Lys146LeufsTer61	Likely pathogenic
7	3.5 year/M	Developmental delay, ID, squint	X-linked ID, Billuart type	*OPHN1*	Hemizygous	NM_002547.3: c.2253_2262delAGCTACTCCC	NP_002538.1: p.Ala752fs	Likely pathogenic
8	1 year/F	Plagiocephaly, squint, delayed milestones, hypotonia	PURA syndrome	*PURA*	Heterozygous	NM_005859.5: c.791G > T	NP_005850.1: p.Trp264Ter	Likely pathogenic
9	3 year/M	Developmental delay, speech delay, microcephaly, happy demeanour, long eyelashes	Schuurs–Hoeijmakers syndrome	*PACS1*	Heterozygous	NM_018026.4: c.607C > T	p.Arg203Trp	Pathogenic
10	2 year/F	Developmental delay, hyperactivity, triangular facies, microcephaly, bushy eyebrows, anteverted nares, hirsutism, clinodactyly, flat feet	ID_Dual diagnosis	*ANKRD11*	Heterozygous	NM_001256183.2: c.1388_1389del	p. Lys463ArgfsTer29	Likely pathogenic
				*EP300*	Heterozygous	NM_001429.4: c.4261dup	p.Tyr1421LeufsTer22	Pathogenic
11	10 year/M	Intellectual disability, ADHD, dysmorphism, microcephaly, thick lips, ring dermoid	ID with dysmorphism	*PITX2*	Heterozygous	NM_001204398.1: c.185–1477C > G	—	VUS
12	4 year/F	Developmental delay, autism, toe walking, stereotypic movements of hands	Atypical Rett syndrome	*MECP2*	Heterozygous	NM_001110792.2: c.433C > T	p.Arg145Cys	Pathogenic
13	8 year/M	Mild intellectual disability, seizures, large ears, coarse facies, synophrys, broad thumbs, club foot	Coffin Siris syndrome	*ARID1B*	Heterozygous	NM_ 020732.3: c.172G > A	p.Ala58Thr	VUS
14	1 year/F	Developmental delay, hypotonia, synophrys, cupped ears, sparse hair, protruding tongue, clinodactyly, hirsutism	Wiedemann-Steiner syndrome	*KMT2A*	Heterozygous	ENST00000534358.8 chr11: g.118473930C > T	p.Ser924Leu	Likely pathogenic
				*AUTS2*	Heterozygous	ENST00000342771.10	p.His535_Thr542del	VUS
15	3 year/F	Seizures, autism, cardiac defect-VSD, growth retardation, microcephaly, dysmorphism	Angelman like syndrome	*GABRA2*	Heterozygous	NM_000807.4: c.455G > A	p.Gly 152Glu	VUS
				*FLNA*	Heterozygous	NM_001110556.2: c.6737C > T	p.Pro2246Leu	Likely pathogenic

## Data Availability

The data that support the findings of this study are available on request from the corresponding author. One *ARID1B* variant details are available on ClinVar_ Accession: SCV001736716.1. The data for other patients are not publicly available due to privacy or financial or ethical restrictions.

## Supplementary Materials

Supplementary Materials Figure 1S: Sanger sequencing for *ARID1B* variant, Figure 2S: Facial profile of Case 9 with *PACS1* gene variant showing subtle dysmorphism and happy demeanor.
