# Correction to “Intellectual Disability and Blended Phenotypes: Insights from a Centre in North India”

**DOI:** 10.1155/crig/9761675

**Published:** 2025-12-31

I. Panigrahi, S. Rao, S. Verma Kumar, D. Kumari, P. Kaur, “Intellectual Disability and Blended Phenotypes: Insights from a Centre in North India,” *Case Reports in Genetics*, 2024, https://doi.org/10.1155/2024/6009569.

In the article, there are errors in the legends for Figures 1, 2, and 1S. The corrected figure legends are as follows:

• Figure 1: Facial features of children with Coffin–Siris syndrome. The facial dysmorphism including coarse facies and hirsutism in child with pathogenic ARID1B variant (Case 2; 1a); another male child with ARID1B variant (Case 13; 1b).
• Figure 2: Facial features showing mild flat facies and prominent cheeks and open mouth in child with PURA syndrome.
• Figure 1S: Sanger sequencing for ARID1B variant c.5467delG (Case 2).

Additionally, the following sentence should be removed from the Results section:

• “Figure 2 depicts facial profile of patient with Coffin–Siris syndrome—patient 2 in Table 1.”

The following sentence in the Results section should also refer to Figure 2, not Figure 1:

• “1‐year‐old female child presented with developmental delay and seizures. Examination revealed plagiocephaly, squint, prominent cheeks, poorly formed nasal columella, inverted V‐shaped upper lip, open mouth (Figure 1), and hypotonia.”

Lastly, the consent statement should be updated to the below:

Written informed consent was taken from the parents for genetic testing, and for photographs from all the participants, and Indian Council of Medical Research (ICMR) ethical guidelines were followed. Written informed consent for publication was obtained from the parents of the identifying images of the children.

We apologize for these errors.

