# Supplementary figures and images for: Intellectual Disability and Blended Phenotypes: Insights from a Centre in North India
Source: Case Rep Genet. 2024 Aug 22;2024:6009569. doi: 10.1155/2024/6009569 (PMC11390182; doi:10.1155/2024/6009569)

## Slide 1
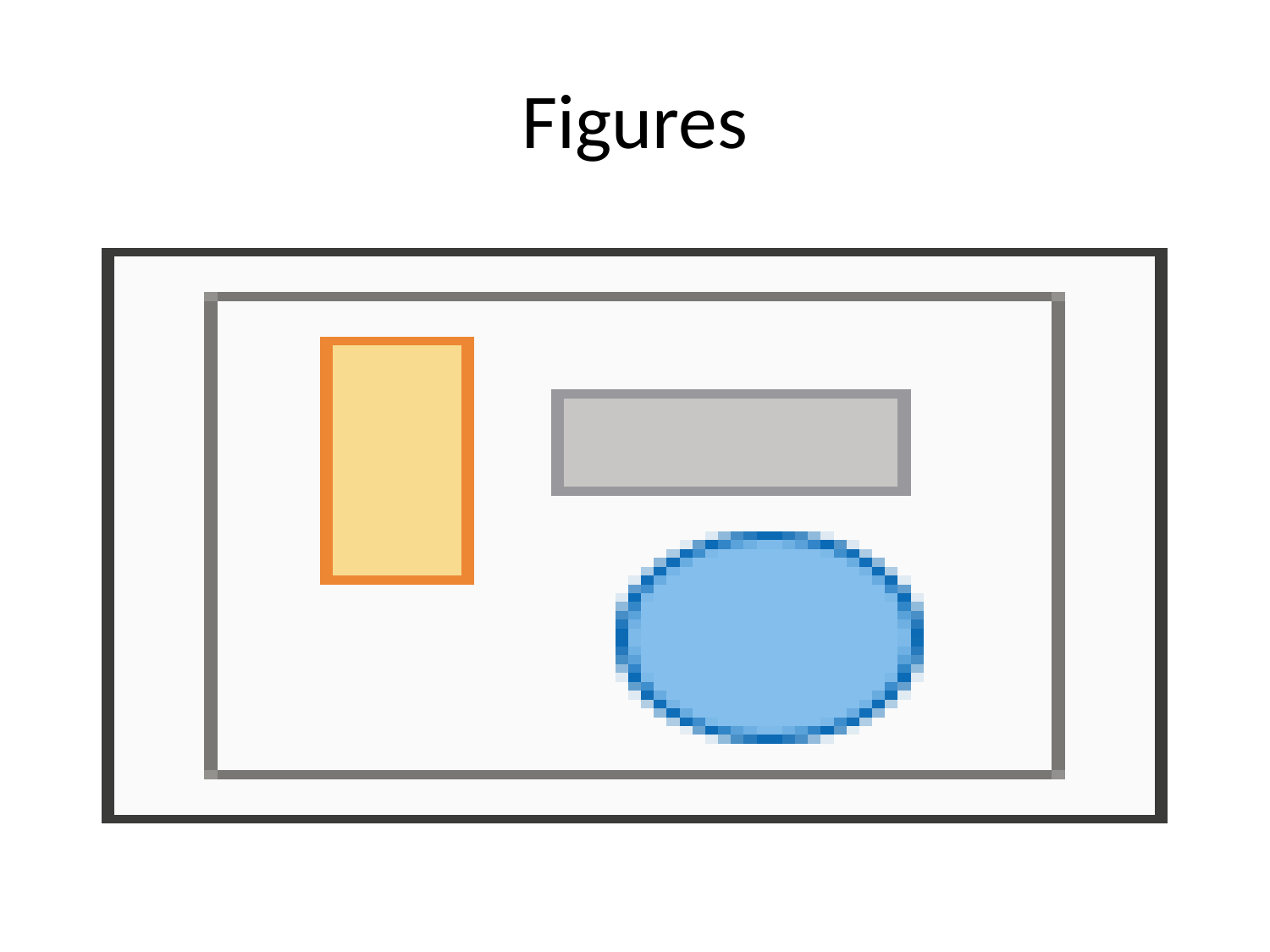

# Figures

## Slide 2
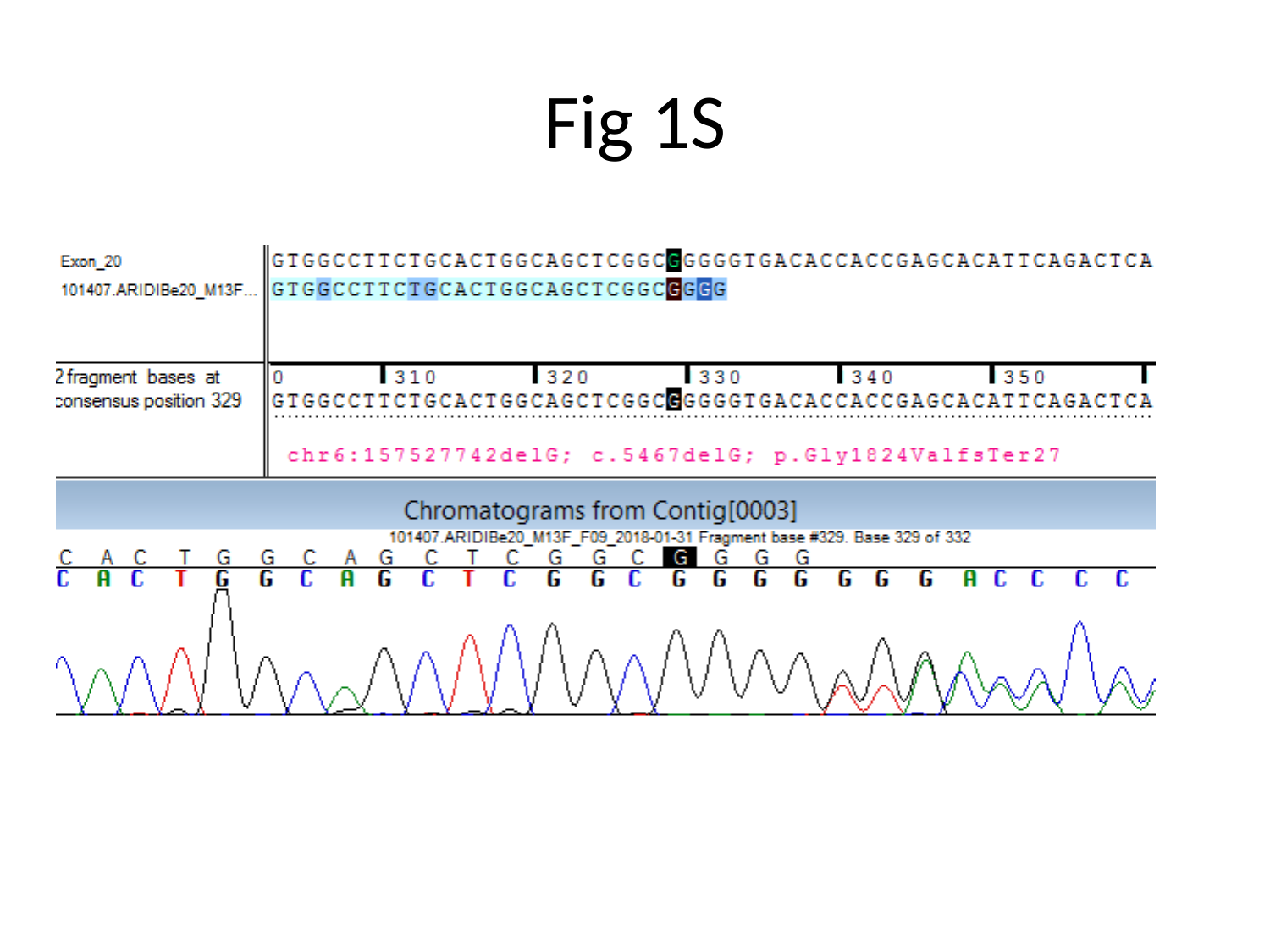

# Fig 1S

## Slide 3
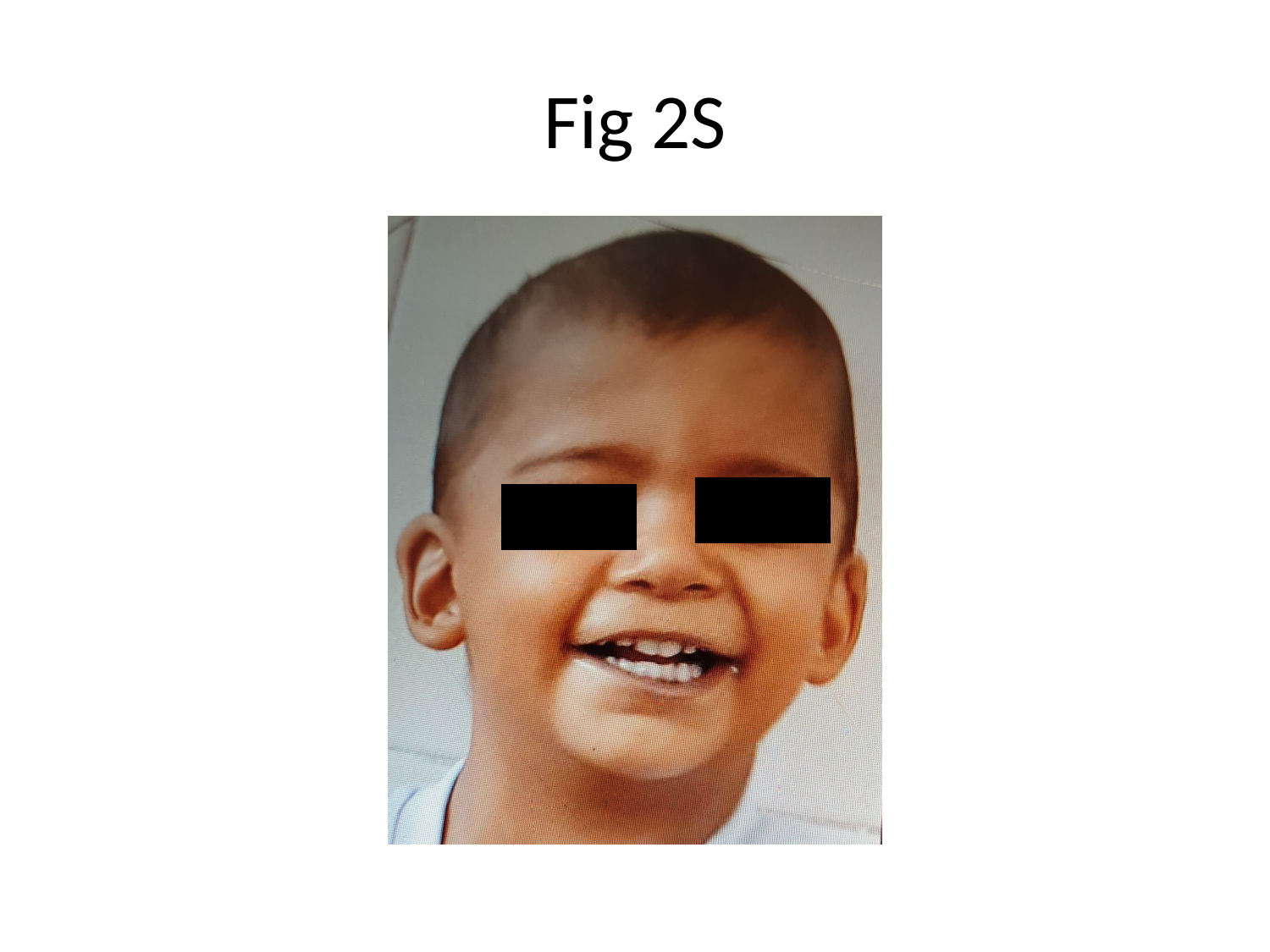

# Fig 2S

Supplement: Supplementary Materials — Figure 1S: Sanger sequencing for ARID1B variant, Figure 2S: Facial profile of Case 9 with PACS1 gene variant showing subtle dysmorphism and happy demeanor. [file 6009569.f1.ppt]
